# HIV diagnoses among people born in Ukraine reported by EU/EEA countries in 2022: impact on regional HIV trends and implications for healthcare planning

**DOI:** 10.2807/1560-7917.ES.2023.28.48.2300642

**Published:** 2023-11-30

**Authors:** Juliana Reyes-Urueña, Gaetano Marrone, Teymur Noori, Giorgi Kuchukhidze, Violetta Martsynovska, Larysa Hetman, Anton Basenko, Stela Bivol, Marieke J van der Werf, Anastasia Pharris, on behalf of the EU/EEA HIV network

**Affiliations:** 1 European Centre for Disease Prevention and Control (ECDC), Stockholm, Sweden; 2 World Health Organization, Regional Office for Europe (WHO/Europe), Copenhagen, Denmark; 3 HIV Diagnosis and Treatment Programs of Public Health Center, Ministry of Health of Ukraine, Kyiv, Ukraine; 4 Alliance for Public Health (APH), Ukraine Cabinet of Ministers’ National Council on HIV/TB (CCM Ukraine), Kyiv, Ukraine; 5 International Network of People Who Use Drugs (INPUD), European AIDS Treatment Group (EATG), Kyiv, Ukraine; 6 Members of the EU/EEA HIV network are listed under Acknowledgements

**Keywords:** HIV infections, epidemiology, population surveillance, migrants, Ukraine, Healthcare

## Abstract

Following Russia’s invasion in 2022, over 4.1 million Ukrainians sought refuge in the EU/EEA. We assessed how this impacted HIV case reporting by EU/EEA countries. Ukrainian refugees constituted 10.2% (n = 2,338) of all 2022 HIV diagnoses, a 10-fold increase from 2021. Of these, 9.3% (n = 217) were new diagnoses, 58.5% (n = 1,368) were previously identified; 32.2% had unknown status. Displacement of Ukrainians has partly contributed to increasing HIV diagnoses in EU/EEA countries in 2022, highlighting the importance of prevention, testing and care.

Over 4.1 million people born in Ukraine sought refuge in European Union/European Economic Area (EU/EEA) countries since Russia’s invasion of Ukraine in February 2022 [1,2]. In 2019, HIV prevalence among the general population in Ukraine was estimated to be 0.9–1.0%, over five times the prevalence in the EU/EEA (0.17%). An estimated 25% of people living with HIV in Ukraine were undiagnosed and 87% of those linked to care were on antiretroviral therapy (ART) [3]. To inform HIV prevention, testing and care programmes, we aimed to assess the impact of the arrival of people born in Ukraine diagnosed with HIV in EU/EEA countries on HIV trends and to describe the characteristics of cases reported in 2022.

## HIV diagnoses in EU/EEA countries

In 2022, 22,995 HIV diagnoses were reported by 30 EU/EEA countries. The majority of those diagnosed were men (70.1%, n = 16,114). Of cases with known mode of transmission (n = 16,718), 46.3% (n = 7,535) were attributed to heterosexual contact and 45.8% (n = 7,457) to sex between men, while 6.0% (n = 963) were attributed to injecting drug use (IDU), 1.6% (n = 252) to mother-to-child transmission (MTCT). Overall, 48.9% (n = 11,103) of those diagnosed in 2022 in EU/EEA countries were migrants, defined as originating from outside of the country in which they were diagnosed [4].

## Impact of diagnosis in people born in Ukraine on HIV trends in the EU/EEA

The notification rate of HIV cases in the EU/EEA in 2022 was 5.1 per 100,000 population, a 30.8% increase compared with 2021 (3.9/100,000 population), but a decrease of 3.8% compared with 2019 (5.3/100,000 population) [4]. People born in Ukraine accounted for 10.2% (n = 2,338) of all HIV diagnoses reported in EU/EEA countries in 2022, a 10-fold increase compared with 2021 (Table 1).

**Table 1:** Number and percentage of HIV cases among people born in Ukraine reported by EU/EEA countries, 2013–2022 (n = 3,380)

Year of reporting	Number of HIV cases among people born in Ukraine	Percentage of HIV cases among people born in Ukraine of all people diagnosed with HIV	Total number of HIV diagnoses reported by EU/EEA countries
2013	67	0.2	28,064
2014	71	0.3	28,075
2015	86	0.3	27,783
2016	86	0.3	27,300
2017	88	0.3	26,693
2018	109	0.4	24,608
2019	137	0.6	24,307
2020	175	1.0	17,714
2021	223	1.2	18,673
2022	2,338	10.2	22,995

EU/EEA: European Union/European Economic Area.

Data are from The European Surveillance System (TESSy) and were extracted on 5 October 2022. All 30 EU/EEA countries reported data for the full period of 2022, however some countries routinely update historical data in future data uploads, and so 2022 numbers could be adjusted slightly in future reporting rounds.

In order to assess the impact of population movement because of the war in Ukraine on HIV trends in EU/EEA countries, cubic splines were used to visualise monthly median HIV diagnoses overall, by gender and by mode of transmission, to compare trends with and without cases born in Ukraine from January 2013 to December 2022 (10 months post-onset of the war). From this analysis, 41,283 HIV reported cases were excluded because of lack of information regarding country of birth or month of diagnosis.

**Figure 1:**
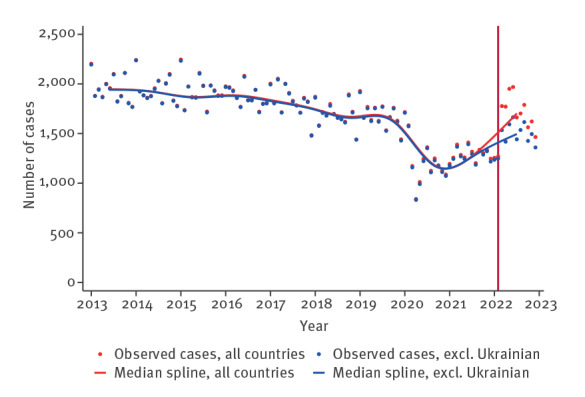
Trends in monthly HIV reported diagnoses in EU/EEA countries, including and excluding HIV cases among people born in Ukraine, January 2013–December 2022 (n = 204,929)

Figure 1 shows a convergence in the number of HIV diagnoses when cases born in Ukraine are included or excluded from the trend reported for all EU/EEA countries until the beginning of the war in Ukraine. After this event, HIV cases born in Ukraine significantly contribute to the overall upward trend.

EU/EEA: European Union/European Economic Area.

The total HIV diagnoses reported during the period were 204,929. Of these, HIV cases among people born in Ukraine totalled 3,183.

Each month, we calculated the median number of diagnosed cases for both the overall number of cases and the number of cases excluding HIV cases among people born in Ukraine. To capture the non-linear trend in both groups, we applied cubic splines for optimal representation.

The vertical red line indicates February 2022, when the war in Ukraine began.

**Figure 2:**
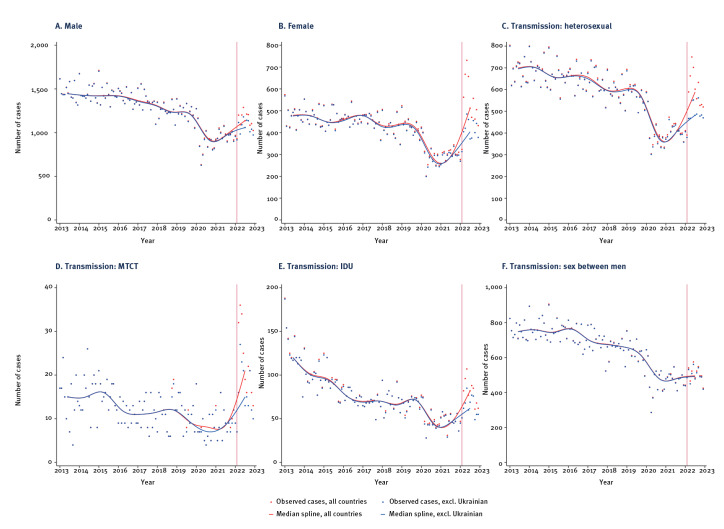
Trends in monthly reported HIV diagnoses in EU/EEA countries, including and excluding HIV cases among people born in Ukraine, by sex and mode of transmission, January 2013–December 2022 (n = 204,929)

In a sub-analysis, a similar impact is observed among women and various transmission modes, including heterosexual, IDU and MTCT (Figure 2). However, the effect is less pronounced in cases where transmission occurred through sex between men.

EU/EEA: European Union/European Economic Area; IDU: injecting drug use; MTCT: mother-to-child transmission.

The vertical red line indicates February 2022, when the war in Ukraine started.

A. Cases among men overall (n = 151,768) and men born in Ukraine (n = 1,385).

B. Cases among women overall (n = 51,807) and women born in Ukraine (n = 1,795).

C. Cases among heterosexual people overall (n = 70,841) and heterosexual people born in Ukraine (n = 1,513).

D. Cases from MTCT overall (n = 1,553) and people born in Ukraine (n = 72).

E. Cases among IDU overall (n = 9,341) and people born in Ukraine (n = 227).

F. Cases among men who have sex with men overall (n = 77,287) and those born in Ukraine (n = 236).

The median number of HIV diagnoses in EU/EEA countries among people born in Ukraine before January 2022 and those diagnosed from February 2022 were compared using a Wilcoxon rank-sum test to assess differences. The median monthly number of HIV diagnoses in EU/EEA countries from people born in Ukraine increased significantly from eight (2013–21) to 173 (2022) overall (results not shown). Among women, the number increased from three to 102, and among men from four to 75. The median also increased significantly in all transmission groups: heterosexual (3 to 71 diagnoses), sex between men (1 to 6 diagnoses), IDU (0 to 13 diagnoses), and MTCT (0 to 4 diagnoses) (p < 0.001).

## New HIV diagnoses and previous positive diagnoses in people born in Ukraine in 2022

The number of HIV cases reported in 2022 among people born in Ukraine by gender, age, mode of transmission, CD4^+^ T-cell count, AIDS status, and ART status are described in Table 2. Diagnoses were categorised based on whether the HIV diagnoses were new (first-time diagnoses in 2022), previous positive (diagnoses made abroad or in another setting before 2022) or unknown.

**Table 2:** Epidemiological and clinical characteristics of HIV diagnoses in people born in Ukraine reported by EU/EEA countries in 2022 (n = 2,338)

Characteristics	New diagnoses		Previous positive diagnoses^a^		Unknown		Total^b^	
	n	%	n	%	n	%	n	%
Total	217	9.3	1,368	58.5	753	32.2	2,338	100
Gender								
Women	136	62.7	845	61.8	464	61.6	1,445	61.8
Men	81	37.3	521	38.1	289	38.4	891	38.1
Unknown	0	0	2	0.1	0	0	2	0.1
Male-to-female ratio	0.6		0.6		0.6		0.6	
Age (years)								
Median age (IQR)	39.6 (0–74)		39.9 (4–71)		38.6 (0–66)		39.5 (0–74)	
< 15	3	1.4	45	3.3	17	2.3	65	2.8
15–19	2	0.9	17	1.2	13	1.7	32	1.4
20–24	9	4.1	26	1.9	26	3.5	61	2.6
25–29	14	6.5	91	6.7	54	7.2	159	6.8
30–39	79	36.4	441	32.2	267	35.5	787	33.7
40–49	81	37.3	547	40.0	290	38.5	918	39.3
≥ 50	29	13.4	198	14.5	84	11.2	311	13.3
Unknown	0	0	3	0.2	2	0.3	5	0.2
Mode of transmission								
Sex between men	13	6.0	78	5.7	12	1.6	103	4.4
Heterosexual transmission (men)	31	14.3	209	15.3	67	8.9	307	13.1
Heterosexual transmission (women)	93	42.9	602	44.0	172	22.8	867	37.1
Injecting drug use	11	5.1	129	9.4	17	2.3	157	6.7
Mother-to-child transmission	2	0.9	61	4.5	15	2.0	78	3.3
Other modes	3	1.4	17	1.2	0	0	20	0.9
Unknown	64	29.5	272	19.9	470	62.4	806	34.5
CD4^+^ T-cell count (cells/μL)								
Median (IQR)	359 (0–4,162)		654 (6–2,091)		507 (1–1,510)		595 (0–4,162)	
< 200	68	31.3	49	3.6	14	1.9	131	5.6
200 to < 350	34	15.7	80	5.8	13	1.7	127	5.4
350 to < 500	19	8.8	136	9.9	9	1.2	164	7.0
≥ 500	44	20.3	504	36.8	28	3.7	576	24.6
Unknown	52	24.0	599	43.8	689	91.5	1,340	57.3
Clinical characteristics								
AIDS	41	18.9	36	2.6	60	8.0	137	5.9
On ART^c^	NA	NA	682	49.9	54	7.2	736	31.5

AIDS: acquired immunodeficiency syndrome; ART: antiretroviral therapy; EU/EEA: European Union/European Economic Area; IQR: interquartile range; NA: not applicable.

^a^ Previous positive cases are defined as an HIV diagnosis made either abroad or in another setting within the reporting country on any occasion before the current year of reporting. Some countries report previous positive HIV cases as they enter, re-enter or re-engage with the care system in the reporting country.

^b ^The proportion of total HIV diagnoses is based on the total number of diagnoses reported, while for newly diagnosed and previous positive diagnoses, the proportion is calculated within their respective row categories.

^c ^Information on country where previous positive HIV cases were diagnosed is not available. However, following discussions with the HIV network and countries receiving a significant number of HIV cases born in Ukraine, it has been ascertained that a majority of people seem to have been diagnosed in Ukraine before the onset of the war.

The majority of cases in both the new and previous positive diagnoses groups were women (n = 136, 62.7% and n = 845, 61.8%, respectively) with a similar mean age (39.5 years; IQR: 0–74). Heterosexual contact was the primary transmission mode for all groups (n = 1,174, 50.2% overall, 76.6% with known transmission mode), with a higher proportion among new diagnoses. Injecting drug use accounted for (n = 157) 6.7% overall, and 10.2% in those with known transmission mode, notably higher among previous positive diagnoses (n = 129, 9.4% overall, 11.8% with known transmission mode). Mother-to-child transmission represented 3.3% (n = 78) overall, and 5.1% in those with known transmission mode, with a higher proportion among previous positive diagnoses (n = 61, 4.5% overall, 5.1% with known transmission mode). The proportion of diagnoses reported with a CD4^+^ T-cell count < 350 cells/μL, which may indicate late diagnosis [5], or an AIDS-defining event at diagnosis was higher among newly diagnosed cases (n = 102, 47.0% and n = 41, 18.9%, respectively) compared with previous positive diagnoses (n = 129, 9.4% and n = 36, 2.6%, respectively). Notably, 49.9% (n = 682) of people categorised as previous positive diagnoses were already on ART at the time of reporting.

## Discussion

In the EU/EEA, HIV diagnoses rose for the first time in a decade in 2022. This increase is partially attributed to post-COVID-19 recovery in health services, testing and surveillance activities [6]. Additionally, the large displacement of people from Ukraine to EU/EEA countries led to a 10-fold increase in HIV diagnoses among people from Ukraine between 2021 (n = 223) and 2022 (n = 2,338). People diagnosed in EU/EEA countries coming from Ukraine comprise two distinct groups: those who were previously diagnosed (most cases, 58.5%), and those diagnosed for the first time (9.3%). The epidemiological profile of both groups reflects the demographics of those most able to travel from Ukraine, primarily women and children [7].

These results have important implications for HIV prevention, testing, treatment, stigma-reduction and surveillance in EU/EEA countries. Prevention that is accessible to new migrant populations, including pre-exposure prophylaxis for HIV (PrEP) as well as needle and syringe programmes and drug treatment for people who use drugs are crucial, especially given that refugees have well-documented vulnerabilities that may incur higher HIV acquisition risk [8].

Among cases with known data on previous diagnosis, the prevalence of late HIV diagnosis (47.0%) and AIDS (18.9%) among newly diagnosed people underscores the urgent need for tailored early testing and linking to care in host countries. This is particularly important because of the known higher HIV prevalence and high undiagnosed fraction of people living with HIV in Ukraine [3]. Enhancing indicator condition testing, antenatal screening and community testing in line with World Health Organization (WHO) and European Centre for Disease Prevention and Control (ECDC) guidelines will help to ensure early diagnosis and linkage to care [9].

Approximately 50% of those who were previously diagnosed with HIV were already on ART at the time of reporting, whereas it is unknown if the other 50% were on ART given data reporting issues. It is important for healthcare services in the host countries to promptly link people with a known HIV diagnosis to treatment and care programmes as soon as possible after arrival both to support individual health and to reduce the risk of increased viral load, which could lead to onward transmission [10].

Considering the proportion of women among Ukrainian cases, EU/EEA countries should review policies for antenatal screening, delivery and post-partum breastfeeding support for HIV-positive women to avoid transmission from mother to child. With a higher proportion of children among those diagnosed with HIV from Ukraine, additional services may be needed from paediatric care services in EU/EEA countries [8]. People who use drugs and have HIV also need access to harm reduction programmes and opioid agonist treatment (OAT) [11,12].

Refugees from Ukraine, similarly to other migrant populations, may fear stigma and discrimination when disclosing their HIV status or risk behaviours or may experience difficulty navigating healthcare services, all of which can impact service uptake [8]. Barriers to HIV prevention, testing and treatment services in host countries, such as language, concerns about stigma and healthcare access must be addressed for all migrant populations, including people from Ukraine [13].

These data highlight the need for enhanced surveillance and monitoring of new HIV diagnoses among people from Ukraine, applicable to other migrant populations as well. The inclusion of country of birth data has been consistent within the HIV surveillance dataset throughout the entire 10-year period. While completeness of this variable exhibits some variation among countries and across different periods within individual countries, the fluctuations are not substantial. A limitation of the analysis is the potential underestimation of people born in Ukraine and lack of full data on previous diagnosis status of these cases. Overall, 11.8% of cases reported by EU/EEA countries in 2022 lacked information on country of birth [4] and 32.2% of cases born in Ukraine lacked information on previous positive diagnosis. Including these variables, as well as CD4^+^ T-cell count at diagnosis and transmission mode in reporting systems, and improving their completeness will enhance our understanding of HIV trends over time as well as characteristics and needs of those newly diagnosed with HIV, providing crucial information to guide public health and healthcare service planning.

## Conclusion

The displacement of people from Ukraine has partly contributed to the increasing trend of HIV diagnoses in EU/EEA countries in 2022. The diagnoses in 2022 include a substantial number of women and previous positive diagnoses, as well as some being new diagnoses, often associated with high rates of late diagnosis. A comprehensive response should prioritise unhindered access to HIV prevention, including PrEP, needle exchange programs and OAT, along with easily accessible testing, prompt diagnosis, linkage and early initiation of ART.
